# Digital navigation as an access barrier: waiting times, equity, and universal health coverage in a digitalizing health system

**DOI:** 10.3389/fpubh.2026.1810870

**Published:** 2026-06-05

**Authors:** Eva Memmel, Lukas Kerschbaumer

**Affiliations:** 1 Center for Social & Health Innovation, MCI, Innsbruck, Austria; 2 Department Nonprofit, Social and Health Management, MCI, Innsbruck, Austria

**Keywords:** access to healthcare, digital divide, digital health equity, health service coverage, qualitative health research, universal health coverage (UHC), waiting times

## Abstract

**Background:**

Universal Health Coverage (UHC) aims to ensure equitable access to timely and appropriate healthcare without financial hardship. Under persistent capacity constraints, access is increasingly governed through non-financial mechanisms such as waiting times. Digitalization is widely promoted to improve efficiency and sustain UHC, yet its equity implications remain contested.

**Methods:**

This qualitative study examines how digitalization reshapes healthcare in Tyrol, Austria, a health system with near-universal health insurance coverage and urban-rural disparities. Drawing on 222 in-depth interviews conducted between 2020 and 2025, the analysis triangulates perspectives from individuals experiencing access vulnerabilities related to financial or digital access constraints, contrast cases with greater access resources, and healthcare experts. Data were analyzed using inductive qualitative content analysis with systematic cross-group comparison.

**Results:**

Digitalization does not eliminate waiting times but redistributes the burden of waiting. Digital tools such as online appointment booking and teleconsultations compress delays and reduce coordination effort for users with sufficient digital skills, connectivity, and institutional trust. For others, limited digital literacy, material and accessibility constraints, language barriers, and reliance on intermediaries intensify uncertainty and prolonged time-to-care. Under capacity pressure, digital navigation becomes a key determinant of effective access, transforming waiting from a temporal delay into a socially stratified burden. These dynamics are particularly pronounced in mental healthcare and in rural settings.

**Discussion:**

In a near-universal healthcare system, digitalization operates as an access-governance mechanism rather than a neutral upgrade. By conditioning access on digital navigation capacities, digitalization produces stratified waiting trajectories despite formal universality of coverage. The pandemic exposed and accelerated these dynamics without resolving the underlying inequalities on which they rest. For UHC implementation, equity depends not only on coverage and financing, but on whether individuals can convert digital access pathways into timely care. Equity-sensitive digital transformation therefore requires multi-channel access, supported navigation, accessibility-by-design, and institutional capacity for mediation to prevent digitalization from amplifying health inequalities and undermining health service coverage.

## 1 Introduction

Universal Health Coverage (UHC) is a central aspiration in global public health and a core pillar of current health policy reforms. At its core, UHC seeks to ensure that all people can access needed, quality health services without financial hardship, while advancing equity in access and outcomes (1, 19). Yet across health systems, the realization of UHC is increasingly shaped by sustained system pressures–workforce shortages, demographic ageing, rising demand for complex chronic care, fiscal constraints, and the growing frequency of crises–such that formal coverage does not automatically translate into timely and appropriate care (2–4). In this context, access is often governed not only through benefit design and cost-sharing, but through non-financial rationing mechanisms such as waiting times, gatekeeping, and complex navigation demands.

Although welfare states differ in how they finance and organize care, a common challenge across regime types is the translation of entitlement into effective access under scarcity (5). Liberal systems tend to expose access more directly to ability to pay, while social-democratic systems reduce many socioeconomic barriers through universal service provision. Corporatist and social health insurance systems, including Austria, achieve near-universal insurance coverage but often retain stratified access conditions through occupational segmentation, supplementary private insurance markets, and uneven capacity across sectors and regions (6–8). Across contexts, these institutional arrangements shape whether UHC is realized as equitable, timely care in practice–especially for groups facing intersecting constraints such as low socioeconomic position, disability, older age, limited language proficiency, and residence in peripheral areas.

Against this backdrop, digitalization is increasingly promoted as a key lever for sustaining UHC under pressure: improving coordination, expanding access pathways, and compensating for shortages without proportional expansion of physical infrastructure (9, 10). At the same time, a growing body of research suggests that digital transformation can reproduce or amplify inequities when access depends on unevenly distributed resources–stable devices and connectivity, digital literacy, accessibility, trust, and the availability of human support (11–14). For UHC implementation, this raises a critical question: when health systems increasingly rely on digitalized access pathways, who can convert these pathways into timely care–and who is left to absorb the costs of delay, uncertainty, and administrative burden? (15).

This study addresses this question in Tyrol (Austria), a high-income, near-universal health system with pronounced urban–rural gradients and persistent capacity constraints. Using qualitative triangulation across participants experiencing access vulnerability related to financial or digital access constraints, a contrast group with comparatively high access resources, and healthcare experts, the study examines digitalization as an access governance mechanism under scarcity. We show that digital tools can compress delays for some users by lowering search and coordination demands, while simultaneously externalizing navigation work and intensifying uncertainty for others. In this way, digitalization does not eliminate waiting; it reshapes how waiting is experienced and by whom–turning digital navigation into a determinant of time-to-care despite formal universality of coverage. By identifying the conditions under which digital pathways function as equity safeguards versus stratifying mechanisms, the findings contribute to UHC debates on equity-sensitive digital transformation and the design of sustainable, multi-channel access systems.

## 2 Background

“Equity is the absence of unfair, avoidable, or remediable differences among groups of people” (16). Rather than implying a uniform distribution of resources, equity aims to achieve comparable outcomes by allocating resources according to differing needs and preconditions (17, 18). This normative understanding is reflected in the principles of Universal Health Coverage (UHC), which emphasize just and equitable access to healthcare and seek to remove structural barriers to health services (19). Such barriers include extended out-of-pocket (OOP) payments that disproportionately disadvantage low-income households, as well as institutional arrangements that condition access to care on health insurance affiliation (20, 21). Although Austria achieves near-universal insurance coverage, with approximately 98% of the population insured (22, 23), its healthcare system can be characterized as a system of stratified, horizontal solidarity, combining income-related risk pooling with contribution-based entitlements structured along occupational lines (6, 8, 24). Healthcare access is primarily organized through compulsory social health insurance, whereby entitlement to services is linked to the payment of contributions determined by employment status and income (8, 25). This institutional design embeds solidarity across insured individuals with comparable contribution obligations, while simultaneously differentiating access conditions and benefit structures between insurance schemes, such as special arrangements for public servants and the coexistence of various public social insurance providers. Despite this solidaristic framework, OOP payments continue to account for approximately 16% of total health expenditure, indicating a persistent financial access component within the system (22, 26). In addition to the statutory social health insurance system, Austria permits a supplementary private insurance market, allowing insured individuals to purchase additional coverage, such as preferential access and shorter waiting times for selected treatments (6). Access to supplementary private insurance is unequally distributed, as such coverage is not financially affordable for all population groups and is therefore closely associated with income and socioeconomic status (27). As a result, approximately 38% of the Austrian population holds supplementary private health insurance, reflecting a substantial yet socially selective expansion of coverage beyond the statutory system (28).

Within this stratified institutional context, waiting times should not, in and of themselves, be interpreted as indicators of poor quality or inefficiency in healthcare provision. Rather, within universal health systems, waiting times are commonly conceptualized as a regulatory mechanism to filter out medically unnecessary treatments–that is, interventions not strictly required to address existing illness causing tangible suffering (29)–and to allocate limited resources according to clinical urgency (30). Within this normative logic, access to healthcare at the system level is intended to be primarily determined by medical criteria rather than financial capacity (31–33). At the individual level, however, effective access may still depend on non-medical factors, particularly health literacy, which influences individuals’ ability to navigate care pathways, articulate health needs, and manage waiting processes (34). Despite the normative framework of UHC, Austria has experienced a marked increase in OOP payments and supplementary private insurance contracts over the past decade (27, 35, 36) driven by patients’ unwillingness to accept long waiting times in the public sector. Waiting periods in both intramural and extramural care are substantially longer for individuals without supplementary private insurance; for example, waiting times for orthopedic surgeries are approximately 9 weeks shorter for patients who pay privately (6, 37, 38). These dynamics are closely intertwined with growing structural challenges in public healthcare provision. Between 2015 and 2019, the number of private general practitioners increased by 32%, while the number of private specialists rose by 64% (39). The resulting decline in physicians working in the public sector, combined with a high proportion of practitioners approaching retirement age and the continued expansion of private provision, raises significant concerns regarding the long-term sustainability of equitable access to healthcare in Austria (22, 37, 40). These systemic inequalities may translate into delayed care for individuals who are unable to afford (timely) private treatment, increasing the risk of prolonged illness, poorer health outcomes, delayed return to work, and heightened income insecurity (31, 33, 41).

Against this backdrop of increasing privatization pressures and capacity constraints within the public healthcare system, digitalization has been increasingly promoted as a potential solution to improve efficiency, alleviate workforce shortages, and enhance access to care (9, 10). Digital tools such as telemedicine, electronic health records (ELGA), and online appointment scheduling are expected to streamline care delivery and reduce access barriers, particularly in underserved and rural regions such as parts of Tyrol. In principle, such tools may help patients identify available appointments more quickly, reduce administrative friction, and bypass bottlenecks in the system (42). However, evidence suggests that digital health solutions may also unintentionally reproduce or exacerbate existing inequalities if underlying social and structural conditions are not adequately addressed (11, 12). Older adults and marginalized groups (e.g., individuals with disabilities, people with lower incomes or in precarious employment) are less likely to have reliable access to digital infrastructure or to possess the literacy required to engage effectively with digital health services or to navigate complex services structures. Amid ongoing digital transformation, these challenges are commonly conceptualized under the term digital divide, which refers to inequalities exceeding basic physical access to devices and connectivity. The concept also encompasses disparities in digital skills, competencies, and the ability to translate digital access into meaningful social and economic advantages. Such inequalities influence a wide range of social domains, including educational attainment, labor market participation, access to healthcare services, and civic engagement (14, 43). With a continuous rise in digitalization within governments and associated service provision (44) the digital divide poses a challenge for effective access to care beyond formal entitlement (45). In Tyrol, rural areas face slower digital uptake and more limited resources for implementing digital health initiatives, further widening regional disparities (46). As a result, the digital divide has emerged as a salient social determinant of health, generating new forms of exclusion for older adults, migrants, and low-income groups who may lack the necessary digital skills, infrastructure, or resources to benefit from digitalized healthcare services (12, 13, 47, 48).

The interaction of traditional access barriers and digital exclusion thus gives rise to a double barrier to healthcare access. Marginalized groups–particularly individuals with low socioeconomic status, older adults, migrants, and residents of rural regions such as Tyrol–may face cumulative disadvantages: longer waiting times within the publicly funded healthcare system are combined with reduced capacity to effectively use digital health tools that might otherwise facilitate faster or alternative access to care. Under these conditions, digitalization does not automatically mitigate existing inequities; instead, it risks reinforcing and amplifying stratified access patterns by disproportionately channeling efficiency gains toward socially and economically advantaged groups (46, 49, 50). Understanding how waiting times and digital access barriers interact is therefore crucial for assessing whether digital health transformation supports equity or inadvertently undermines the principles of UHC. By triangulating perspectives from services users experiencing access vulnerabilities (such as socioeconomically disadvantaged users), frontline health and social service staff, and healthcare experts in Tyrol (Austria), this study shows how digitalization under capacity constraints can convert digital navigation into a determinant of time-to-care, producing stratified waiting and cumulative disadvantage even within a near-universal health system. In light of these intersecting dynamics, the present study examines how digital exclusion interacts with existing access barriers to shape healthcare access for marginalized populations in Austria, with a particular focus on regional disparities in Tyrol.

## 3 Methods

This study employed a qualitative research design to examine how waiting times, institutional arrangements, and digitalization shape equitable access to healthcare in Austria. Data collection began during the COVID-19 pandemic, a period in which healthcare access, education, and social services became simultaneously constrained and increasingly mediated through digital infrastructures. The pandemic is explicitly treated as a structural “stress test” that rendered existing inequalities and access mechanisms particularly visible, rather than as a temporary disruption of otherwise stable systems. To reflect on these circumstances, contrastive data from 2024 and 2025 were included. Fieldwork was conducted in the Austrian federal state of Tyrol. Tyrol constitutes a particularly relevant setting for examining healthcare access due to its pronounced urban–rural gradient and geographically fragmented service landscapes, which affect both provider availability and the implementation conditions for digital health initiatives.

### 3.1 Study period and iterative approach

Data collection took place between March 2020 and December 2025 as an ongoing, iterative research process in which preliminary analyses continuously informed subsequent data collection (51). While the core interview guideline remained stable across the study period to ensure comparability, the guide was refined in December 2023 to include a more specific focus on digital barriers. This refinement was prompted by the prominence of digitalization and digital exclusion in earlier interviews and aimed to deepen the analysis of access mechanisms related to digital infrastructures, skills, and navigation practices, without altering the overarching research focus. Given the temporal span of the material, the analysis allows a careful analytic distinction between pandemic-specific disruptions during acute phases of COVID-19 and the persistence or transformation of access barriers in the post-pandemic period.

### 3.2 Participants, sampling, and recruitment

**Figure 1:**
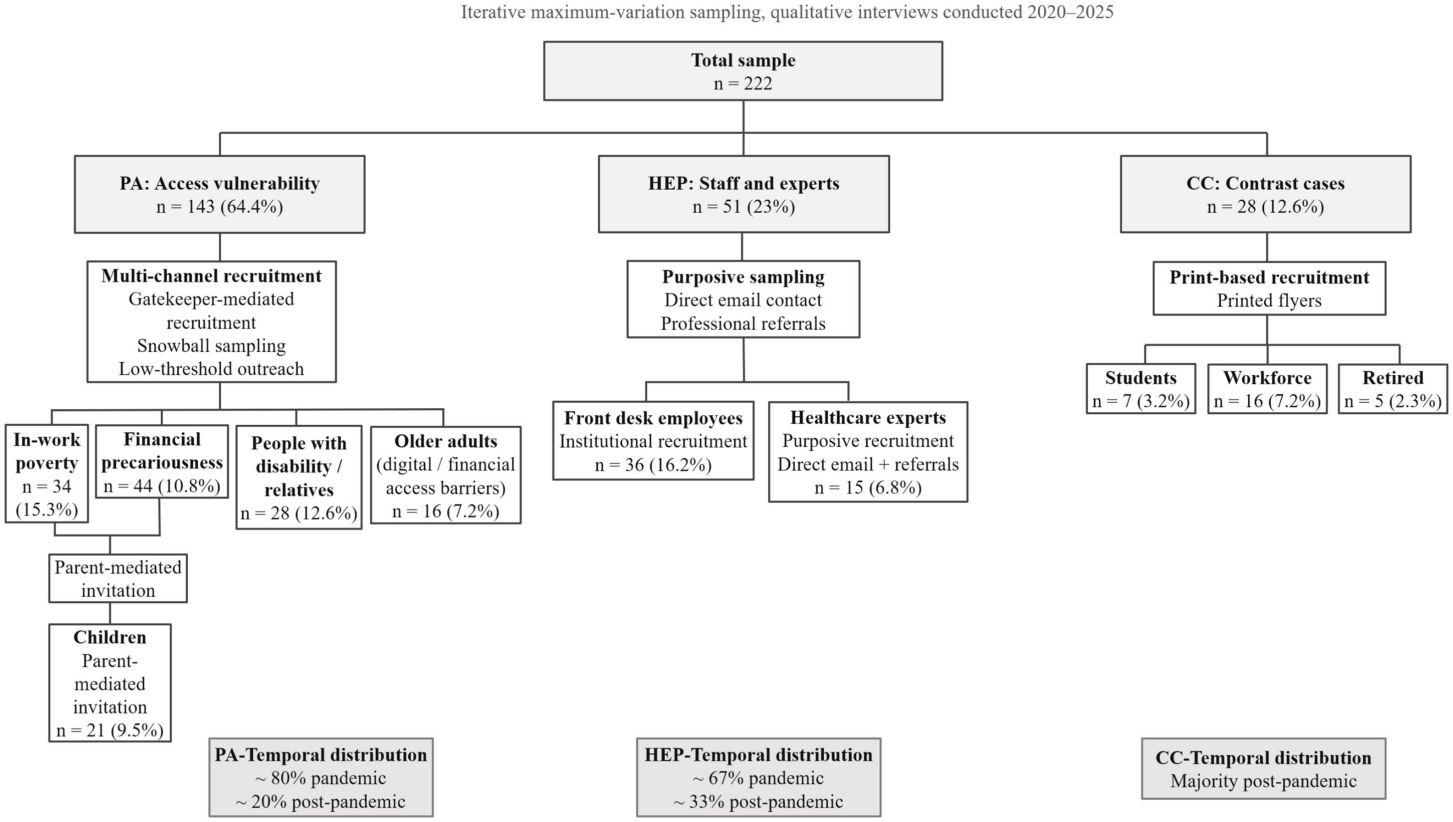
Recruitment pathways and final sample composition.

Overall, the study comprises 222 qualitative interviews, including 143 interviews with individuals experiencing financial precariousness or other access vulnerabilities, 36 interviews with frontline health and social service staff working in consultancies, administrative bodies, institutions, or associations, 15 interviews with healthcare experts, and 28 contrast cases (Table 1). Sampling aimed for maximum contrast in order to capture a wide range of perspectives across social positions, institutional roles, age groups, and vulnerability constellations (52). More specifically, sampling followed an iterative maximum-variation strategy designed to capture heterogeneous positions of vulnerability rather than statistical representativeness. The sample was constructed to include variation across social position, age, disability, and institutional role, and combined affected participants with contrast cases and professional actors to enable systematic comparison across groups (Figure 1).

Recruitment pathways differed across subgroups and were aligned with the analytic purpose of the study. Participants experiencing access vulnerability were recruited primarily through a multi-channel strategy combining gatekeeper-mediated recruitment via counselling agencies and social service organizations, snowball sampling, and targeted low-threshold outreach. Gatekeeper-mediated recruitment facilitated access to vulnerable groups and helped establish trust, while snowball sampling enabled access to harder-to-reach participants through existing social networks (85, 86). Low-threshold outreach involved direct recruitment in everyday support settings such as food banks, where potentially underrepresented groups could be approached outside formal service or research contexts (53). This broader stratified approach was used in particular for participants in work poverty, individuals in financially precarious situations, and people with disabilities or related access vulnerabilities. Children and adolescents were not recruited directly. Instead, participating parents were asked whether they would be willing to pass on an invitation to their children to participate in the interview study. This parent-mediated pathway was chosen for practical and ethical reasons and enabled the inclusion of children’s perspectives while ensuring that recruitment occurred through already established relationships of trust and awareness. Both, children and parents were asked for their consent. During the iterative recruitment process, older participants were initially underrepresented relative to their analytic relevance for questions of digital access, service navigation, and healthcare use. Recruitment was therefore increasingly directed toward older adults in later phases of fieldwork in order to strengthen this dimension of comparison.

Healthcare experts were recruited through purposive sampling, mainly via direct email contact and supplemented by professional referrals (54, 55). Experts were defined as individuals with professional responsibility for designing, implementing, or managing healthcare services, or with privileged access to decision-making processes (56). Contrast cases were recruited as an analytically distinct comparison group and were defined by the absence of current financial distress. Their inclusion was intended to provide comparative insight into how access pathways, waiting, and digital navigation are experienced when participants have greater socioeconomic and organizational resources. In temporal terms, the distribution of interviews also varied somewhat across groups: within the vulnerability-related subgroups, a smaller proportion of interviews was conducted in the post-pandemic phase (20%), whereas a larger share of contrast cases was recruited post-pandemically. This distribution was considered analytically in order to distinguish pandemic-specific disruptions from more persistent access mechanisms.

Recruitment of all non-expert participants was additionally supported through project websites and printed materials, including flyers and posters placed at selected community locations. At the same time, reliance on gatekeepers may introduce selection bias, as some influence over sample composition can shift to institutional actors (87, 88). To counteract this risk, gatekeeper-mediated recruitment was combined with snowball sampling and low-threshold outreach. Snowball sampling enabled access to hard-to-reach groups and leveraged trust within existing social networks, while the combined use of multiple recruitment strategies reduced the likelihood of confinement to narrow subgroups (57). Recruitment and analysis proceeded iteratively until adequate information power was achieved within subgroups and for the main analytic categories, rather than aiming for statistical representativeness (58). Saturation was operationalized at two levels: within each subgroup, data collection continued as long as interviews yielded new themes or access-related mechanisms, and sampling proceeded to further subgroups until no substantively new patterns emerged in cross-group comparison. Decisions to cease sampling within a group were therefore not based on predefined quotas but on the analytic judgement that additional interviews were unlikely to alter the emerging category system – a judgement revisited iteratively as analysis and data collection proceeded in parallel (59).

**Table 1:** Overview of the sample.

Sample	*N*	%
Children (age 9–16) (financial challenges)	21	9.5
In work poverty	34	15.3
Individuals in financial precarious situation	44	19.8
People older than 50 (financial challenges or access vulnerabilities)	16	7.2
People with disabilities and relatives (financial challenges/access vulnerabilities)	28	12.6
Number of people in precarious situations or with access vulnerabilities (PA)	**143**	**64.4**
Front Desk Employees in Social, Public and Care Institutions	36	16.2
Healthcare experts	15	6.8
Number of Frontline and healthcare expert participants (HEP)	**51**	**23.0**
Contrast cases: Students	7	3.2
Contrast cases: Workforce	16	7.2
Contrast cases: retired	5	2.3
Number of contrast cases (CC)	**28**	**12.6**
Total sample	**222**	**100.0**

Throughout the analysis, the category PA is used as a shorthand for participants facing financial precariousness or experiencing challenges in accessing social or healthcare services related to digital barriers and other forms of access vulnerability. For readability, this group is referred to in the following sections as participants experiencing access vulnerability (PA). This group includes children and adolescents aged 9–16 years (*n* = 21), working poor individuals (*n* = 34), adults in financially precarious situations including unemployed persons (*n* = 44), people older than 50 years (*n* = 16, including 5 retirees), and people with legally recognized disabilities as well as their relatives (*n* = 28), where financial challenges, digital barriers, or service access constraints were present. Analytically, PA denotes limited capacity to absorb administrative, temporal, and digital access burdens under conditions of constrained service provision, rather than poverty alone.

The category HEP, used as a shorthand for health and expert participants, includes participants working in the health and social sector, including physicians, therapists, and front desk employees in social, public, and care institutions. The category CC refers to the contrast group and includes individuals not currently facing financial precariousness, including seven students, 16 working individuals aged 22–67 years, and five retired individuals. The coding system describes interviews in the format *Interviewnumber_Group_Sex_Age* for the PA and CC groups and does not include the age variable for participants from the HEP group.

### 3.3 Data collection

Data were generated through semistructured interviews with healthcare experts and for all other participants following biographical-narrative interviews combined with problem-centered techniques (60, 61). This approach enabled participants to situate experiences of access to social and healthcare, waiting times, and digital barriers within broader life trajectories, while allowing systematic exploration of institutional pathways, coping strategies, and perceived inequities. To minimize exclusion, interviews were offered in multiple formats. Depending on public health regulations and participant preferences, interviews were conducted in person, by telephone, or via online platforms such as Zoom, Skype, or Microsoft Teams (62, 63). Participants could choose the interview mode most suitable to their circumstances; experts more frequently preferred online interviews. During lockdown phases, all procedures complied with public health regulations. The increasing reliance on digital communication during this period was thus both a methodological consideration and part of the phenomenon under investigation. Interviews typically lasted between 45 and 60 min. For participants with disabilities, interview procedures were adapted to accessibility needs. All study information and consent materials were provided in easy-to-read versions. Interviews were conducted in paced, simplified language and included options for breaks when needed. These interviews were supported by a social care professional experienced in disability services and augmentative and alternative communication. Their involvement facilitated accessible participation and enabled the collection of in-depth qualitative accounts of the lived experience of individuals with disabilities (64). Given the heterogeneity of disability experiences, no single operational definition was applied; inclusion followed international, European, and national legal definitions encompassing physical, mental, intellectual, and sensory impairments lasting longer than 6 months (65, 66).

### 3.4 Analytic strategy and triangulation

All interviews were transcribed verbatim and analyzed using inductive qualitative content analysis (67). Coding was conducted using meaning units and refined iteratively through constant comparison within and across participant groups. MAXQDA was used to support data management, coding, and retrieval. During analysis, particular attention was paid to how participants described waiting not merely as elapsed time, but as a sequence of actions, efforts, and uncertainties associated with accessing care, especially under digitally mediated conditions. The analysis explicitly pursued triangulation across actor positions by integrating perspectives from individuals experiencing access vulnerabilities, frontline health and social service staff and healthcare experts, and the contrast group (68). This triangulation enabled systematic comparison between lived experiences, institutional practices, and professional assessments of system functioning, supporting the identification of access-related mechanisms operating across social positions and system levels. To ensure rigor in the coding process, a proportion of the material was independently coded by a second team member during the initial phase of analysis, and this procedure was repeated at the onset of coding for each subpopulation. Discrepancies were discussed in regular team meetings until consensual agreement on category definitions and boundaries was reached (67, 69). These meetings also provided an opportunity to reflect on the emerging category system across participant groups and informed iterative decisions about whether further sampling was needed or whether adequate information power had been achieved (58, 70).

The analytic purpose was to examine intersectional vulnerability and to identify mechanisms through which access barriers emerge, persist, or change across social positions and institutional contexts (71). Digitalization-related barriers were not treated as group-specific phenomena; instead, themes were examined across the entire sample, with attention to variation in manifestation and consequences. Themes captured experiences of access, including waiting times, navigation across services, use of digital access routes, and perceived effort, uncertainty, and persistence required to obtain care. Importantly, waiting times were not introduced as a direct interview topic but emerged through participants’ narratives of access. Biographical reconstructions were used to contextualize accounts within broader life courses and to trace processes linking socioeconomic vulnerability, digital barriers, waiting times, and health-related outcomes (72).

### 3.5 Ethical considerations and reflexivity

The research was conducted in accordance with established ethical standards for qualitative research studies. All participants received detailed information about the study and provided informed consent prior to participation, with the option to withdraw at any time (73–75). For minors and people with disabilities, consent procedures were designed to acknowledge independence while ensuring guardians’ awareness and approval; both minors/ people with disabilities and their legal guardians were asked to provide consent. Confidentiality and anonymity were ensured throughout data collection, analysis, and reporting. Adult participants received €50 per interview, and children of participating parents received €80 within the group of financially challenged. Experts were excluded from receiving financial incentives. Ethical approval was granted by the responsible university ethics committee (reference number: 20220403).

The authors are affiliated with the Center for Social and Health Innovation and the Department Nonprofit, Social and Health Management at MCI Innsbruck and conducted the study in Tyrol, the regional context under investigation. Their proximity to the field facilitated access to participants and institutions and supported a nuanced understanding of local healthcare and social service structures. At the same time, this proximity may also have shaped expectations regarding inequality, precariousness, and the role of digitalization in access to care. Reflexive attention was therefore paid throughout data collection and analysis to how researcher positioning might influence interview dynamics, thematic emphasis, and interpretation. Analytically, this was addressed through iterative comparison across participant groups, the inclusion of contrast cases and expert perspectives, and continuous discussion of emerging interpretations within the research process.

## 4 Results

### 4.1 Healthcare access under capacity constraints

This section examines how healthcare access is organized and experienced under conditions of persistent capacity constraints in a near-universal healthcare system. Drawing on interviews with healthcare professionals, service users, and frontline staff, the findings first analyze the social regulation of waiting times as a core mechanism for managing scarcity. The section then explores how digitalization has been introduced as a complementary strategy to reorganize access without expanding physical infrastructure, reshaping both institutional processes and patient responsibilities. Together, these findings illustrate how access to care is increasingly mediated through a combination of waiting, navigation capacity, and digitally structured pathways.

### 4.2 Capacity constraints and the social regulation of waiting times

Across interviews with healthcare professionals, waiting times were described as a structurally embedded feature of healthcare provision rather than merely a symptom of inefficiency. Within this logic, waiting times were understood to serve a regulatory function by prioritizing urgent cases, discouraging medically unnecessary interventions, and allowing self-limiting conditions to resolve without clinical treatment. As one interviewee emphasized, waiting was viewed as a deliberate and meaningful threshold: “[...] a waiting time is also a very important and sensible threshold filtering out of trivial consultations is essentially achieved through waiting times” (129_HEP_M). This framing illustrates that waiting times were not primarily perceived as system failure, but as an accepted and purposeful mechanism for managing demand and allocating care under constrained capacity. This understanding was further reinforced by the same participant who explicitly framed waiting times as an alternative to financial rationing: “Either I make the system fee-based, which creates a social barrier […] or I introduce waiting times” (129_HEP_M). This reasoning reflects a core UHC logic: waiting operates as a form of non-financial rationing–a distributive mechanism intended to preserve formal financial accessibility while managing scarcity–shifting rationing into the temporal domain. From this perspective, waiting is not an unintended by-product of scarcity, but a deliberate governance choice aimed at avoiding direct price barriers–albeit one that redistributes access costs in the form of time, uncertainty, and delayed care. As another professional explained, “A lot of things work themselves out. Our bodies are pretty good at healing” (101_HEP_F). In this sense, time itself was framed as a therapeutic and allocative resource–one that can reduce unnecessary diagnostics and overtreatment, while presuming patients’ capacity to endure and navigate delay.

Concurrently, the aforementioned group of participants emphasized that waiting times become harmful when they delay diagnosis or treatment for conditions in which timing is clinically decisive. Particularly severe consequences were reported in mental healthcare, oncology-related diagnostics or pain management. In these exemplary areas, delayed access was associated with symptom deterioration, chronicity, reduced quality of life, and, in extreme cases, life-threatening outcomes. The potential severity of delayed access was illustrated by one interviewee referring to time-sensitive pathways and rare but critical escalation: “Funnel chest, for example, can develop and heart problems can occur. In the worst case there was even a death because the [patient] did not get an appointment” (124_HEP_M). Especially in psychiatric and psychological care, professionals stressed that access barriers intersect with limited “windows of readiness” (105_HEP_M): if timely appointments are unavailable, care trajectories may break down entirely, with individuals disengaging from services for extended periods.

Waiting times were not experienced uniformly across medical disciplines. Psychiatry, psychotherapy, psychosomatic care, diagnostic imaging, gynecology, and dermatology were repeatedly identified as high-bottleneck areas, where long waiting times emerged as a symptom of structural scarcity. This scarcity was mainly attributed to workforce shortages and reimbursement structures that inadequately compensate counselling-intensive and diagnostically complex care, in fields where longer consultation durations are intrinsic to effective treatment. In contrast, general inpatient pediatric care was perceived as comparatively accessible, although highly specialized pediatric services–such as developmental neurology–were characterized by extensive waiting times. These differences indicate that waiting times are not simply ‘natural’ outcomes of demand but reflect institutional and financing arrangements that shape how scarcity is distributed across fields of care.

Regional disparities further intensified unequal access experiences. Healthcare experts consistently described a pronounced urban–rural gradient, particularly in Tyrol’s geographically fragmented landscape. As one participant summarized, “With regard to equal opportunities, I would say that there is the urban-rural divide. In rural areas, it is more difficult to access healthcare […]” (123_HEP_F). In remote valleys and peripheral districts, specialist care often required long travel distances, while provider absences could immediately translate into access gaps due to limited “backup” options (101_HEP_F). Under these conditions, waiting times became not only longer but also less predictable–amplifying uncertainty for individuals with limited flexibility, mobility, or financial resources.

Importantly, interviewees emphasized that waiting times do not affect all patients equally. One professional explicitly situated these unequal waiting experiences within broader social stratification rather than individual behavior or system malfunction:

*Equal opportunities start much earlier. If you have money, you have better chances. And whether someone has money is not only determined by individual performance, but by social background and family circumstances. We can try to compensate within the system, but this inequality will remain.”* (124_HEP_M).

This reflection highlights a shared understanding among professionals that healthcare systems can attenuate, but not fully offset, inequalities rooted in socioeconomic position. In this sense, waiting times do not merely reflect clinical prioritization or organizational capacity, but intersect with pre-existing social advantages that shape who can absorb, endure, or circumvent delays.

While formally oriented toward medical urgency, effective access depended on individuals’ capacity to navigate complex care pathways, identify available services, manage administrative requirements, and–crucially–on access to supplementary private insurance and the ability to persist through delays. As one professional noted, “There is certainly a difference between people with supplementary insurance [and without]. But I would say that individual factors are also certainly important” (101_HEP_F). Patients with higher health literacy, flexible employment conditions, stronger informal networks, or the financial means to pursue private alternatives were better positioned to cope with or circumvent waiting, whereas disadvantaged groups faced higher risks of postponed or foregone care. In this sense, waiting times functioned as a socially differentiated access mechanism that redistributes the burden of system scarcity onto patients. However, another interviewee highlighted that unequal waiting times are not only a matter of speed, but of access to effective care: “If I can afford a private doctor, of course I will have a shorter waiting time. But the crucial question is: waiting time for what? It’s not just about getting an appointment in three days, but about getting an appointment where something [adequate] actually happens” (129_HEP_M). This distinction underscores that waiting time functions not only as a quantitative but also qualitative access mechanism, shaping not only how quickly care is accessed, but whether access leads to meaningful diagnosis or treatment.

Against the backdrop of stretched service capacities and pronounced regional disparities, digitalization was widely perceived as a key strategy to mitigate access barriers without expanding physical infrastructure. Digital tools were expected to improve coordination, facilitate appointment allocation, and partially compensate for limited provider availability–particularly in rural areas. Reflecting this perceived potential, one professional emphasized: “And, yes, from a medical point of view, I believe digitalization is a very important component there too” (101_HEP_F). At the same time, interviewees described a qualitative shift in how access to care is organized: as booking, documentation, and information exchange increasingly move into digital environments, responsibility for navigating availability, monitoring waiting times, and coordinating care is progressively transferred to patients. Accordingly, digitalization was not described as a uniform solution, but as a context-dependent modifier of access conditions under persistent capacity constraints.

### 4.3 Digitalization under capacity pressure: efficiency gains and shifting responsibilities

Where digital tools were perceived as well implemented and aligned with clinical workflows, healthcare professionals reported tangible efficiency gains. Iterative improvements to internal clinical software tools were described as making work “faster and easier,” yielding a clear “efficiency benefit” in everyday routines (154_CC_M_48). From a system perspective, interviewees involved in hospital digitalization emphasized leaner processes, improved transparency of documentation, and enhanced patient safety when information could be accessed across professional roles and locations. As one expert summarized, “with a digital documentation or digital solutions in general [the] quality in the healthcare sector is increasing” (173_CC_M_30).

On the user side, online appointment booking emerged as a particularly salient access facilitator. Participants repeatedly highlighted its low threshold and time-saving nature, emphasizing that appointments could be secured quickly, “in two minutes”, without navigating telephone queues (162_CC_F_25). At the same time, efficiency alone was not sufficient to ensure perceived access. While online systems reduced friction and saved time, participants emphasized that digital channels did not always provide the sense of security and confirmation associated with direct human interaction. Under conditions of urgency or uncertainty, users often preferred analog contact, as it offered immediate reassurance that an appointment was successfully arranged. This ambivalence highlights the legitimacy of parallel access routes: digital tools can enhance efficiency, while non-digital channels remain crucial for trust, confirmation, and situational urgency.

Teleconsultations further contributed to maintaining access and continuity of care, particularly during periods of restricted mobility. Several healthcare professionals reported the normalization of remote formats in clinical practice, including “online bookings […] and also consultations through video” (170_CC_F_67). However, digital care was consistently framed as complementary rather than substitutive. Interviewees emphasized that remote consultations worked best when embedded in hybrid care pathways that included regular in-person encounters. In some settings, informal rules emerged–such as conducting “two consults online, one at least in person”–to ensure that relevant physical or psychosocial cues were not missed and that clinicians could “see what’s happening […] in real life” (172_CC_F_32).

At the same time, participants described substantial ambivalence toward digital transformation. Not all digital tools reduced workload or improved access. In some cases, digital pilots increased administrative burdens, particularly when systems were poorly integrated or insufficiently adapted to clinical routines. One hospital’s pilot of an electronic temperature chart was described as increasing documentation time: “If they wrote a temperature chart by hand, it took about ten minutes, but digitally it took around half an hour per patient” (152_CC_F_26), ultimately leading to resistance and a return to analog processes. Similarly, users reported difficulties with unstable or confusing public platforms, including “constant error messages” (151_CC_F_22) in government applications, which required them to troubleshoot independently via search engines or alternative tools. These experiences exemplify how the broader shift of responsibility to patients translates into concrete forms of additional work, rather than simply faster access.

Crucially, interviewees emphasized that digital-by-default strategies risk intensifying existing inequalities if not accompanied by adequate institutional support. Frontline staff described how digital transitions increased demands on workers and could not replace relational, in-person services without sufficient staffing, training, and time. In practice, staff frequently had to complete online forms on behalf of clients, illustrating that usability and navigability–rather than mere technical access–constituted core barriers. Whether digitalization reduced or reinforced access inequalities thus depended less on the existence of digital tools per se than on implementation quality, interoperability, and the availability of human support.

### 4.4 Multi-channel access as an equity strategy: trust, urgency, and geography

In response to both the opportunities and limitations of digital tools, participants consistently described a situational and conditional use of access channels. Rather than replacing traditional modes, digital systems were embedded within multi-channel strategies that balanced efficiency, urgency, and perceived reliability. Online booking was predominantly used for planned, non-urgent needs, while telephone contact remained the preferred option for urgent scheduling or complex situations. As one participant explained, “If I need an appointment quickly […] I call. And if I only need something in a few months […] then I book it online.” (154_CC_M_48). Trust emerged as a central dimension shaping channel choice. Some users explicitly preferred telephone contact because it provided immediate confirmation “that an appointment worked,” whereas online requests left them uncertain about whether a slot was truly secured (152_CC_F_26). Maintaining parallel access routes was therefore widely perceived not as redundancy but as an equity mechanism. This conditional use indicates that multi-channel strategies remain essential even for digitally capable users, because the perceived reliability of pathways varies by urgency and complexity rather than by digital skill alone. Interviewees cautioned that older adults and individuals with limited digital experience “really struggle” with multi-step online processes; without simplification, digitalization risked becoming an additional barrier rather than an enabler (154_CC_M_48).

Finally, the effectiveness of digital access strategies was strongly mediated by geography and time. In high-demand urban settings, platforms that aggregated appointment availability could materially shorten waits by revealing openings that remained inaccessible via conventional channels. For individuals with limited time or financial resources, online scheduling reduced travel and coordination costs–“having quick online access saves me trip costs across the city” (156_CC_M_23). In rural contexts, however, dedicated support infrastructures were thinner. While *ad hoc* local initiatives existed to assist older adults with digital access, sustained provision was limited, underscoring the continued necessity of analog options and human assistance to prevent exclusion. “In my hospital it’s fast and reliable. But in lower settings, in smaller cities, […] they had a lot of issues during COVID-19 and still have some of them now” (172_CC_F_32).

Taken together, these findings indicate that digitalization reshapes healthcare access not by eliminating scarcity, but by reorganizing how scarcity is managed and by whom. While digital tools can reduce friction and waiting for some, they simultaneously introduce new prerequisites for effective access–such as digital literacy, device availability, and the capacity to navigate complex platforms. The following section therefore examines how these digitally mediated access mechanisms intersect with social stratification, focusing on financial constraints, poverty, digital literacy, and cumulative disadvantage across different population groups.

### 4.5 Digital stratification and cumulative disadvantage

While digital tools can reduce administrative friction and reorganize access under conditions of capacity constraints (Section 4.1), their benefits are not distributed evenly. The following findings show that digitalization does not operate as a neutral efficiency mechanism within UHC systems, but becomes an access-governance-mechanism. It reshapes how access is navigated under capacity constraints and becomes embedded in existing social, economic, and institutional inequalities. Across interviews, material constraints, uneven digital competencies, and accessibility gaps shaped who could benefit from telehealth, online appointment systems, and increasingly digitalized welfare and healthcare procedures. In practice, digital access was not simply an ‘additional channel’, but a socially patterned precondition for making use of time-saving options, securing information, and maintaining continuity across care and welfare pathways. Across these accounts, waiting was rarely described as a single, clearly bounded delay; instead, it emerged as a sequence of attempts, coordination tasks, and uncertainties that accumulated unevenly depending on digital skills, resources, and available support.

#### 4.5.1 Preconditions of digital access: material resources and skills

Across interviews, economic precarity consistently emerged as a primary barrier to digitally mediated health and social services. Participants did not describe digital exclusion primarily as a matter of preference or resistance, but as a costed condition of participation–one that required stable devices, functioning software, and, crucially, continuous connectivity. Low-income households repeatedly reported insufficient devices, reliance on shared equipment, and unstable or absent internet contracts that undermined participation in health and social services. The dilemma was captured succinctly: “But what good is a rented laptop if you have no internet connection […] or internet provider” (18_PA_F_14). This statement illustrates an important mechanism in the data: device access alone does not constitute effective access. When connectivity is missing or unreliable, ‘having’ a device becomes a hollow form of inclusion–formal access without functional usability. In these accounts, the internet contract appears as a hidden infrastructure of rights-claiming: without it, online appointment systems, e-government procedures, and telehealth options remain technically available but practically unreachable.

Material constraints were particularly visible in family contexts. One participant described the strain of providing minimal digital capacity for multiple household members: “We had to buy TWO laptops and that was really a problem” (23_PA_M_45A). This quote points to how digitalization can convert social participation into a household-level investment requirement. When schooling, welfare procedures, and health-related communication shift online, families are forced into difficult trade-offs between devices/connectivity and other essential expenditures. In this sense, digitalization can operate as a budget pressure amplifier, intensifying stressors that are themselves relevant to health and economic security.

Frontline accounts echoed these constraints and emphasized that economic precarity compounded digital exclusion and narrowed access to telehealth and online services, particularly for those lacking stable connectivity or the means to maintain contracts. Importantly, participants described how these barriers could become acute during crisis periods. During the pandemic, online-only procedures became a de facto gatekeeper to essential supports; when internet contracts were terminated to reduce costs, applying for benefits and accessing services became markedly more difficult for economically vulnerable groups. These accounts portray a shift from digitalization as optional convenience to digitalization as an obligatory interface–and thus as a new point where poverty translates into administrative and health disadvantage.

The compounded effect was visible in education as well: “Mostly I used my mobile phone at home, so I don’t have a computer or tablet at home” (55_PA_F_15). Here, the mobile phone appears as a substitute infrastructure–a minimal access device that enables some participation, but often at the cost of functionality, privacy, and ease of completing complex tasks. Participants linked device scarcity and unstable connectivity to disrupted learning, widening disadvantages, and additional stressors–dynamics that in turn spill over into health and care capacity (e.g., stress, uncertainty, reduced ability to manage procedures).

Alongside material resources, participants repeatedly linked digital exclusion to age and educational background, describing older adults and people with lower formal education as particularly likely to struggle with basic digital tasks and online procedures: “Many older people never learned how to deal with this” (151_CC_F_22). Several accounts framed these difficulties not as marginal gaps but as a fundamental threshold of participation in contemporary service landscapes: “It’s like a new form of illiteracy. What used to mean not being able to read and write now means not being able to use a computer” (06_PA_F_55). By equating digital skills with basic literacy, participants highlighted that digitalization establishes a new access requirement that is unevenly distributed along biographies shaped by education, occupational history, and opportunities for digital learning. As services move online, this unequal distribution of competencies becomes consequential: it shapes who can independently search, apply, upload, confirm, and follow up–and who becomes dependent on intermediaries.

Geography further moderated access. Professionals observed an urban-rural gradient, suggesting that digital pathways may mitigate travel burdens for some, but cannot resolve structural shortages where providers are scarce and analog safety nets are weak. Socioeconomic position additionally shaped perceived value: individuals confident in navigating platforms and with flexible schedules emphasized convenience–“digitization also saves a lot of time” (163_CC_M_37)–yet they also cautioned against over-reliance on AI-mediated information and highlighted ethical concerns such as bias, especially in health contexts. Overall, these results show that digital access operated less like a neutral channel and more like a stratifying resource, unevenly distributed and unevenly convertible into tangible access gains.

#### 4.5.2 (Digital) navigation as a sorting mechanism: efficiency, waiting, and circumvention

Beyond devices and connectivity, effective access depended on the ability to navigate multi-step, often confusing platforms. A young adult described encountering “constant error messages” on public applications and having to troubleshoot through search engines and other tools. These accounts illustrate that digitalization can shift problem-solving labor onto users: instead of a completed procedure, individuals encounter a requirement to diagnose platform failures, seek informal knowledge, and persist through uncertainty. For those with time, confidence, and digital routines, such troubleshooting can be irritating but manageable; for those under time pressure, with limited literacy, or with low trust in online systems, it becomes a deterrent that interrupts access trajectories.

Participants articulated an explicit equity principle that connects these experiences to distributive justice: “digitalization is great, but it shouldn’t depend on how much money you have. So, the access should be equal because otherwise people like me fall behind easily” (156_CC_M_23). This statement reflects a broader pattern: digitalization was widely recognized as beneficial in principle, but only under conditions that prevent unequal conversion of “efficiency” into advantage. In other words, the data suggest that efficiency is not inherently equitable; it becomes equitable only when the prerequisites for capturing efficiency gains are widely available.

For low-income families, literacy barriers also shifted roles within households. Several parents reported relying on children to complete online applications: “The children filled out the applications” (12_PA_F_51). This quote captures an inversion of competence and responsibility: rather than digital services empowering households, digitalized procedures can reallocate administrative labor to those with the skills–sometimes minors–while exposing parents’ vulnerability and dependence. In some accounts, the outcome was not merely inconvenience but withdrawal from benefits altogether when processes felt inaccessible or overwhelming. Thus, digital navigation becomes consequential not only for healthcare entry points but also for welfare stability–an upstream determinant of health.

Access channel choice reflected differences in literacy, trust, and perceived reliability. While some users relied on online booking for routine needs, many reverted to telephone contact in urgent or complex situations to ensure confirmation and reduce uncertainty. This conditional channel use illustrates that digitalization increases cognitive and coordination demands: deciding when and how to switch channels becomes part of the work of accessing care.

At the same time, participants described how digitally confident patients were better able to navigate availability across providers, use online tools, and thereby partially circumvent long waiting times–an option largely inaccessible to those with limited digital skills: “[…] booking online appointments or checking the opening hours […] And without these digital tools, it would take a lot more time” (156_CC_M_23). In these accounts, digital navigation functions as a sorting mechanism: it accelerates access for those who can search, compare, and persist, while others remain concentrated in slower pathways or forego care. The result is not simply different user experiences, but stratified time-to-care.

#### 4.5.3 Accessibility, language, and frontline mediation: cumulative disadvantage

Accessibility shortcomings–rather than the mere absence of digital tools–were a dominant barrier for people with disabilities. Participants and providers described exclusion stemming from inaccessible technologies and insufficient assistive supports, underscoring that inclusion requires design and usability, not just availability. One participant emphasized interface opacity: “I think there are often websites where I just don’t understand the user interface” (162_CC_F_25). Others described structural inaccessibility: many websites are “not accessible for blind people” (132_HEP_F). Even where “easy-to-read” materials existed, coverage was inconsistent (132_HEP_F). These findings indicate that digitalization can reproduce exclusion when accessibility is treated as an add-on rather than a foundational requirement. Remote formats introduced further barriers when presentation materials were unreadable by screen readers: “with a PPT, I have no chance because I can’t read it” (135_HEP_M). This participant illustrates how exclusion can occur within digital encounters, not only at entry points. When teleconsultations, online information, or hybrid formats rely on inaccessible file types, the interaction becomes closed off–even if the user is otherwise digitally connected. Providers echoed these concerns, cautioning that online booking and digital communication are “super” for “Digital Natives,” but may disadvantage older adults and cognitively impaired people without adequate assistance (105_HEP_M). In practice, usability and navigability–not merely access–constituted central barrier.

Participants described how unmet accessibility needs increased reliance on frontline mediation. When platforms or procedures were too complex, staff and intermediaries frequently had to fill out online forms on behalf of users. This does not eliminate exclusion; it shifts it into a dependence relationship and into an organizational resource problem. Where staff capacity is limited, supported navigation becomes scarce, producing another layer of inequality: access depends on whether relational support is available at the point of need.

Importantly, institutions and service providers did not locate these challenges solely on the side of service users. Across interviews, organizational actors emphasized that the digital transformation of access pathways also constitutes a challenge for staff themselves. In particular, the accompaniment of people with disabilities in digitally mediated tasks increasingly requires personnel to act as digital intermediaries–supporting registration processes, handling authentication, scanning and uploading documents, and navigating complex platforms. This role expansion was described as especially demanding for older staff members, who themselves reported difficulties in keeping pace with rapidly evolving digital tools and procedures. To continue supporting affected individuals under increasingly digitalized conditions, interviewees pointed to the need for targeted further qualification and ongoing training for staff, underscoring that digital inclusion is co-produced at the institutional level rather than achieved through technology alone.

Front-desk staff described how the growing culture of digital ‘self-service’ in welfare and healthcare systems creates new exclusion mechanisms that extend beyond language barriers. In their accounts, insufficient digital competence frequently became the decisive bottleneck–ranging from difficulties navigating websites and understanding procedural logic, to challenges completing application forms and managing required documentation. As one frontline worker explained: “But there is often a lack of basic digital literacy. Just operating a computer or completing a registration process. They usually need a lot of help” (141_HEP_F). This account illustrates how digitalization transforms access into a sequence of technically mediated tasks–registration, authentication, scanning, uploading, and bundling documents–that presuppose a level of digital base education many clients do not possess. When these competencies are missing, access to benefits and services becomes contingent on intensive personal support. In contexts where such support is limited, delayed, or unavailable, digital procedures can lead to interrupted applications, foregone entitlements, and heightened stress–dynamics that are especially consequential when administrative barriers intersect with psychological strain and reduced coping capacity.

Language barriers further intersected with digital exclusion across the care pathway, reinforcing cumulative disadvantage. Limited German proficiency constrained use of digital health and administrative platforms, often available only in German or requiring advanced reading, making migrants and people with limited language proficiency particularly reliant on external assistance for booking, forms, and information: “Online services are often very difficult for people […], especially if there is a migration background” (147_HEP_M). These barriers carried over into clinical encounters, where time-limited consultations compounded communication hurdles:

*Equal opportunities, when it comes to patient care, are certainly worse in health insurance practices... because there are simply a lot of people who do not speak German very well. And it’s simply not possible to have an in-depth conversation in such a short time. In my opinion, this means that patients with a migrant background who speak poor German simply receive poorer medical care. (119_HEP_F).*

In practice, the additional minutes required for interpretation and clarification, that are often not reimbursed, reduced the depth of comprehensive assessment and counselling, reinforcing a dual burden: dependence on intermediaries to navigate digital entry points and constrained communication during care. The data thus point to cumulative disadvantage: accessibility and language barriers shape not only whether people reach care, but also the quality of interaction once care is reached.

These accessibility deficits intersected with broader resource constraints in mental health services. Mental health access emerged as a critical equity domain within this cumulative burden. Families and providers reported long waits, cost barriers, and digital hurdles that led to unmet need during periods of heightened stress and isolation: “What is totally missing are positions for mental health problems. Financially and time-wise, psychotherapy is simply not feasible […]. People wait months for a spot to become available” (149_HEP_F). For households already facing device scarcity and unstable connectivity, online mental health services were often inaccessible, compounding distress and interrupting care pathways when support was most needed.

Taken together, this section shows how the layering of material constraints, accessibility barriers, language limitations, and reliance on frontline mediation can produce cascading effects–delayed care, foregone benefits, and reduced participation–outcomes that are central to population-level inequities in access, prevention, and continuity of care.

### 4.6 The double barrier: interaction of digitalization and waiting times

Taken together, Sections 4.1 and 4.2 show how core principles of UHC–universal entitlement combined with non-financial rationing–are enacted in practice under digitalized conditions. They indicate that digitalization does not merely add an access channel under conditions of scarcity; it reshapes how waiting is navigated and by whom. Across interviews, participants described a double barrier in which (I) limited provider availability increases the value of rapid information and flexible scheduling, while (II) uneven digital prerequisites–such as skills, devices, connectivity, accessibility, language proficiency, and trust–determine who can leverage digital pathways. Under conditions of congestion, this interaction made digital navigation a key determinant of time-to-care, reproducing unequal waiting experiences despite the formal universality of coverage. Building on this interaction, the findings show that waiting was not experienced as a single, discrete delay but as a cumulative burden–one that unfolded through repeated attempts to secure appointments, uncertainty about outcomes, and the coordination work required to navigate fragmented access routes. Digitalization redistributed this burden unevenly: for some, it compressed delays and reduced coordination effort, while for others it intensified exposure to uncertainty, administrative friction, and prolonged time-to-access.

Under capacity pressure, digitally confident users described using online booking systems and digital information channels to identify available appointments and reduce coordination costs, particularly for planned and non-urgent care. Others linked digital routines to reduced time and travel burdens. In contrast, participants experiencing access vulnerabilities (PA) as well as frontline staff described that limited digital skills, unstable connectivity, inaccessible interfaces, or language barriers reduced the ability to use online systems for booking, confirmations, and follow-up. In these accounts, digital exclusion translated into fewer opportunities to search across providers or react to availability, increasing reliance on slower pathways and extending time-to-access. Participants thus described ‘waiting’ not only as time in a queue, but as an unequal burden of repeated attempts, uncertainty, and coordination work–burdens that digital tools reduced for some while increasing exclusion for others.

Furthermore, participants described how digitalization could amplify delays when systems were already strained. Older adults and digitally less confident users were repeatedly described as struggling with multi-step procedures and unclear interfaces: “things are often made more complicated, or you need several steps before you reach your goal” (154_CC_M_48). Under high demand, failed logins, error messages, and uncertain confirmations added time and uncertainty and increased dependence on telephone support or in-person assistance. On the provider side, interviewees emphasized that digital tools did not automatically reduce workload. In some settings, digital documentation and coordination requirements increased time pressure and constrained staff capacity, particularly when professionals were expected to compensate for patients’ navigation difficulties. Under such conditions, digitalization shifted coordination work onto both patients and providers, reinforcing delays and limiting the availability of relational support.

Finally, participants emphasized that uptake of digital formats depended on trust, perceived quality, and clinical suitability. Clinicians cautioned that remote encounters can miss important cues and should be balanced with in-person contact: “on the online consults, we don’t see the full perspective […] we can miss some important sign” (172_CC_F_32). In disability contexts, respondents stressed that “personal advice is necessary” (137_HEP_F). Where access models shifted toward digital-first pathways without robust analog alternatives or supported navigation, participants described compounded delays–particularly in mental healthcare, where capacity shortages were already pronounced: “People wait months for a spot to become available” (149_HEP_F).

Overall, the findings suggest that waiting times and digitalization are co-produced access conditions rather than independent barriers. Under capacity constraints, digital tools enabled some users to reduce waiting by improving access to information and scheduling flexibility. At the same time, uneven digital prerequisites and differential trust concentrated others in slower and more uncertain pathways and increased reliance on overstretched support structures. Participants pointed to potential mitigation measures such as “affordable workshops,” “simple government platforms,” and “subsidies [for] low income people to buy decent devices […] the access should be equal” (156_CC_M_23), underscoring the importance of multi-channel access and supported digital navigation if efficiency gains are to be distributed equitably.

### 4.7 Temporal variation: pandemic overload and post-pandemic persistence

The temporal span of the material suggests that the COVID-19 pandemic should be understood not merely as background context, but as a stress test that exposed and intensified pre-existing access vulnerabilities and institutional bottlenecks. In the acute pandemic phase, access difficulties increased through service disruptions, unclear crisis communication, the rapid expansion of digital and at times effectively online-only procedures, and the transfer of coordination burdens to patients, families, schools, and frontline support organizations. Under contact restrictions, digital access shifted from an optional convenience to a practical precondition for participation in healthcare, welfare, and education. During this period, digitalization also became a central institutional concern, and participants described at least temporary recognition that additional support and resources were needed to enable access, even where newly introduced digital arrangements were poorly adapted to the needs of low-income households, older users, or people with limited digital literacy. The post-pandemic material indicates, however, that this heightened attention did not translate into lasting structural change. Instead, the pandemic accelerated and normalized digitalized access pathways while leaving inequalities in digital literacy, material resources, accessibility, and supported navigation capacity largely intact:

“Digital illiteracy, dealing with technical equipment for the digital world–these are massive challenges. You often hear comments like, ‘Everyone has a smartphone.’ But that’s one thing; Email is another challenge. Governmental systems are still difficult to navigate.” *(142_HEP_F).*

Participants’ accounts suggest that emergency attention to digital exclusion gradually receded, while many of the procedures introduced under crisis conditions remained in place. As a result, many of the same groups that had struggled during the pandemic remained similarly disadvantaged afterward. From participants’ perspectives, the system absorbed the shock only partially. Digital and improvised hybrid arrangements maintained a degree of continuity, but this continuity was achieved less by removing bottlenecks than by shifting substantial coordination and adaptation work onto users, families, and frontline intermediaries: “Actually, I informed myself, no one informed me. I really have to say that. Yes, so there is simply a lack of information from the outside somehow” (15_PA_F_54). Institutions and service providers described these dynamics not only as a service-user problem, but also as a workforce and organizational capacity problem. While some experts emphasized clear benefits of digitalization for efficiency, coordination, and continuity of care, they also stressed that the rapid rollout of digital procedures created immediate demands for staff support, often without sufficient preparation, training, or time. These pressures were especially pronounced among older staff members and in organizations working with people with disabilities, where digital mediation became an additional layer of support work. Importantly, these challenges persisted beyond the acute crisis: digital procedures remained in place, while staffing and resource shortages continued. This created a structural tension, as the very institutions expected to compensate for digital exclusion were often least able to invest in the workforce development, training, and time resources needed to do so. Analytically, the pandemic thus functioned less as a temporary exception than as an amplifier and accelerator of pre-existing stratified access dynamics.

**Key Result: Waiting as a digitally redistributed burden under UHC**

Across interviews, waiting emerged not merely as time spent in a queue, but as a cumulative burden of coordination, uncertainty, and repeated attempts to access care. Digital tools reduced this burden for users with sufficient skills, connectivity, and trust, while intensifying exclusion for others. Under conditions of capacity constraints, UHC implementation increasingly hinges on digital navigation, which becomes a key determinant of time-to-care and produces stratified waiting trajectories despite formal universality of coverage. The pandemic did not create these inequalities anew, but exposed, accelerated, and normalized them by making digital access temporarily indispensable while leaving underlying inequalities and institutional support deficits largely intact.

## 5 Discussion

This study examined how digitalization interacts with existing access barriers in a near-universal healthcare system under sustained capacity constraints, contributing to current debates on how Universal Health Coverage (UHC) is realized in practice. By triangulating the perspectives of participants experiencing access vulnerability (PA), contrast cases, and healthcare experts, the findings demonstrate that digitalization does not function as a neutral efficiency-enhancing layer. Instead, it restructures access by transforming digital navigation into a prerequisite for timely care. Under conditions of scarcity, this produces a double barrier: long waiting times increase the value of rapid information and flexible scheduling, while uneven digital prerequisites determine who can leverage these opportunities. Rather than eliminating waiting, digitalization redistributes the burden of waiting under UHC implementation, compressing delays for some while extending them through navigation work, uncertainty, and repeated coordination for others. The temporal comparison further suggests that the COVID-19 pandemic acted less as a temporary exception than as an accelerator of these dynamics. Crisis conditions intensified digital dependence and accelerated the institutionalization of digital access pathways without a corresponding reduction in underlying inequalities in resources, literacy, accessibility, and institutional support. In this way, emergency adaptations became durable features of stratified access. Conceptually, the study therefore reframes waiting not merely as a neutral temporal delay, but as a socially stratified burden redistributed through digitalization under conditions of scarcity and crisis-accelerated system change. This contribution extends existing research on digital health equity by moving beyond binary notions of inclusion versus exclusion. Rather than asking whether people ‘have access’ to digital tools, the findings show that equity hinges on whether individuals can convert digital opportunities into effective access. Digitalization thus emerges as a conditional access regime whose equity effects depend on material resources, skills, institutional support, and trust (15, 76).

### 5.1 Waiting times revisited: from temporal delay to social exposure

Consistent with prior literature, healthcare experts in this study framed waiting times as a legitimate mechanism for rationing care under constrained capacity, prioritizing urgency and filtering out medically unnecessary consultations (31, 32). However, the findings complicate this normative account by showing that waiting is not experienced as a uniform temporal delay, but as a socially differentiated exposure to uncertainty, effort, and risk. For participants experiencing access vulnerability related to financial or digital access constraints (PA), waiting often entailed repeated attempts to access services, uncertainty about whether care would materialize at all, and a heightened risk of disengagement–particularly in mental health contexts where readiness and continuity are fragile. Participants in the contrast case group often described waiting as a manageable inconvenience, mitigated through flexibility, information-seeking, and the ability to switch providers or pathways. These findings align with research showing that rationing mechanisms, while formally neutral, produce unequal outcomes when they intersect with social resources and health literacy (33, 41). Importantly, this study adds that digitalization intensifies this differentiation by reshaping what it means to ‘wait’: waiting becomes an active process of monitoring, navigating, and coordinating access–activities that are unevenly distributed across social groups (32).

The study shows that digitalization reorganizes healthcare access by redistributing coordination and administrative work. Tasks such as searching for appointments, managing registrations, uploading documents, handling authentication, and resolving technical failures increasingly fall on patients or intermediaries rather than institutions. This redistribution was highly patterned by social position. Individuals from the contrast group described digital tools as resources that enabled them to actively manage access and reduce waiting times. Participants experiencing access vulnerability, especially those with a low socioeconomic position, often lacked the material stability, digital confidence, or cognitive bandwidth required to perform this work, turning digitalization into an additional barrier rather than a facilitator. Healthcare experts corroborated these experiences, noting that digital tools often increase, rather than reduce, overall system workload by shifting coordination tasks to patients and frontline staff. This resonates with critiques of digital-by-default governance, which highlight how efficiency gains are frequently achieved by externalizing labor to users and frontline health and social service staff without reducing structural scarcity (11, 14, 77).

These dynamics are further intensified by regional and infrastructural disparities, particularly along rural–urban lines. In rural and peripheral areas, limited provider availability already translates into longer and less predictable waiting times. At the same time, digital access pathways, which are often promoted as a means to mitigate geographic distance, depend on reliable broadband infrastructure and digital service coverage that are unevenly distributed across regions (78). Where digital infrastructure is weaker, the capacity to use online booking systems, teleconsultations, or digital information channels is correspondingly reduced, even as the need for such tools is greater due to provider scarcity. When these territorial constraints intersect with low socioeconomic position, older age, disability, or limited digital literacy, digitalization risks producing a compounded form of exclusion. In this sense, digital determinants of health–including access to reliable internet infrastructure at the community level–operate alongside individual resources to shape equity in access and outcomes (79). Rather than functioning as equalizing mechanisms for UHC implementation, digitalization in these contexts can amplify existing access barriers by redistributing waiting and coordination burdens onto populations least able to absorb them (14, 28, 78). From a UHC perspective, this underscores that equity-sensitive digital transformation must address not only individual capacities but also regional infrastructural conditions if digital access strategies are to reduce, rather than reproduce, spatial inequalities in time-to-care.

The temporal dimension of the material adds a further layer to this argument. The pandemic did not create these inequalities from scratch, but acted as a stress test that intensified and accelerated them. Under acute crisis conditions, digital access became temporarily indispensable for participation in healthcare, welfare, and education, while the burden of coordination was increasingly shifted onto users, families, and frontline intermediaries. Importantly, the post-pandemic material suggests that this reorganization of access did not recede once restrictions were lifted. Rather, many of the digitalized procedures introduced under crisis conditions remained in place, while the underlying inequalities in resources, digital literacy, and institutional support persisted (49, 80). In this sense, the pandemic appears less as a discrete interruption than as an accelerator of a more durable transformation in how waiting, navigation, and access are organized.

### 5.2 Digital stratification as a cumulative social process

The findings indicate that digital stratification cannot be reduced to a single ‘digital divide’. Instead, it emerges as a cumulative process shaped by interrelated dimensions repeatedly identified in the data, including material resources, digital skills, accessibility, language proficiency, and trust in institutions. These dimensions interact across life domains, linking healthcare access to welfare administration, education, and becoming particularly consequential in care contexts that are highly sensitive to delay and continuity, such as mental health services. For participants experiencing access vulnerability (PA), particularly those facing low socioeconomic positions, digital exclusion often coincided with economic insecurity, unstable housing, and psychological strain, creating feedback loops that intensified vulnerability. These patterns align with evidence showing how socioeconomic insecurity and digital exclusion interact to compound health and social disadvantage (81). Reliance on mobile phones as substitute infrastructure, dependence on children as digital intermediaries, and withdrawal from services when procedures became overwhelming illustrate how digitalization can transform disadvantage into compounded exclusion. These dynamics are also particularly pronounced for people with disabilities, for whom access to digitalized services often depends on proxies such as care professionals or family members. The findings suggest that these intermediaries can themselves become access bottlenecks when they lack sufficient digital training, time, or institutional support. Under conditions of thin staffing and high care workloads, frontline professionals described limited capacity to keep pace with rapidly evolving digital systems, while family members faced similar challenges without formal training or recognition. As a result, digitalization may inadvertently shift access barriers from individuals with disabilities to the very support structures meant to enable inclusion, reinforcing dependency rather than reducing it. Participants’ framing of digital skills as a “new form of literacy” underscores the structural nature of these barriers. As services become digital-by-default, digital competence functions as a foundational capability for effective participation in health and welfare systems–one that remains unevenly distributed along educational, occupational, and generational lines (13, 43, 82).

Taken together, the results support conceptualizing digital exclusion not merely as a technical or administrative issue, but as a social determinant of health. The findings show that digital barriers shape access across interconnected domains–including healthcare, welfare benefits, education–thereby influencing income security, stress exposure, continuity of care, and coping capacity. In care domains that depend on sustained engagement and timely access, such as mental health services, these barriers translate into particularly acute risks of delay. Importantly, the data suggest that digital exclusion operates primarily through indirect pathways, including delayed or foregone care, interrupted benefit claims, heightened administrative stress, and reduced engagement with preventive or mental health services. These mechanisms align with broader public-health frameworks that emphasize how social conditions structure exposure to health risks and access to protective resources (16, 83). By empirically linking digital barriers to cumulative disadvantage across multiple domains, this study strengthens the argument that digital inclusion should be treated as an integral component of health equity strategies rather than as a peripheral innovation issue.

### 5.3 Same system, unequal conversion: a cross-group mechanism

A key contribution of this study lies in demonstrating that all participant groups describe and interact with the same healthcare system, yet experience fundamentally different access trajectories depending on their social position. The inequalities observed do not stem from parallel systems or formal exclusion, but from differential capacities to convert identical institutional conditions into effective and timely care. The table below synthesizes this cross-group mechanism by juxtaposing how digitalization under scarcity is enacted, interpreted, and experienced across groups.

Table 2 synthesizes how digitalized access pathways under conditions of capacity constraints are enacted, experienced, and interpreted across participant groups within the same formally universal healthcare system. It contrasts participants experiencing access vulnerability (PA), participants with comparatively high access resources (contrast cases, CC), and healthcare experts (HEP) across key dimensions of access governance, including digital access, waiting, coordination work, and the role of staff.

**Table 2:** Digital navigation under scarcity: group-positioned access pathways.

Dimension	Poverty affected (PA)	Contrast cases (CC)	Health experts (HEP)
Digital access	Threshold of participation	Navigational resource	Organizational challenge
Waiting	Compounded burden and uncertainty	Manageable delay	Legitimate rationing
Coordination work	Externalized and overwhelming	Strategically managed	Shifted to patients and staff
Role of staff	Essential intermediaries	Occasional support	Increasing workload
Outcome	Delayed or foregone care	Reduced time-to-care	Stratified access acknowledged

Read analytically, the table illustrates that inequality in access does not arise from unequal entitlement, but from unequal capacity to convert formally universal coverage into timely and effective care. Digitalization functions less as a uniform access channel and more as a conversion amplifier: it lowers coordination costs and compresses waiting for some, while externalizing coordination burdens and intensifying uncertainty for others. The table thus makes visible how waiting and digital navigation jointly operate as stratifying mechanisms within Universal Health Coverage implementation under scarcity. For participants experiencing access vulnerability, digital access constitutes a threshold of participation. Failure to meet basic digital requirements–such as stable connectivity, functional devices, and sufficient digital literacy–often results in delayed, fragmented, or foregone care. Waiting, in this context, is not merely a temporal delay but a compounded burden characterized by uncertainty, repeated attempts, and dependence on intermediaries. Digital coordination demand is experienced as overwhelming because it accumulates across domains (healthcare, welfare, education), often under conditions of psychological stress and material insecurity. In contrast, participants with greater access resources are able to mobilize digital tools as navigational assets–monitoring availability, switching providers, and choosing channels and pathways strategically. Here, coordination demands are not eliminated but rendered manageable through higher digital literacy, flexible time resources, and economic buffering capacity, including access to supplementary insurance. Waiting therefore remains present, but is more often experienced as an inconvenience rather than a risk to care continuity. Healthcare experts, in turn, describe digitalization primarily as an organizational challenge. While waiting times retain legitimacy as rationing mechanisms, digital tools simultaneously shift coordination work onto patients and staff, generating a structural tension between efficiency expectations and growing demands for supported navigation. Importantly, healthcare experts explicitly recognized the resulting stratification of access, even as they lacked institutional capacity to fully offset it. Taken together, the table makes visible that inequality arises through unequal conversion of formal entitlement into effective and timely care rather than unequal entitlement. Digitalization intensifies this conversion problem by increasing the value of resources–digital skills, cognitive capacity, time flexibility, and supportive networks–that are themselves unequally distributed. In this sense, digitalization does not simply interact with social inequality; it recalibrates how inequality translates into time-to-care within UHC under conditions of scarcity (15, 76, 84).

From a Universal Health Coverage perspective, these findings underscore that near-universal insurance coverage is insufficient to guarantee equitable access when care is mediated through waiting and digital navigation. Formal entitlement must be complemented by institutional arrangements that enable individuals to translate coverage into timely and appropriate care. Accordingly, the policy implications extend beyond digital infrastructure toward an equity-sensitive digital transformation that embeds safeguards into access design–including multi-channel access, supported navigation as a UHC safeguard, accessibility-by-design as a default rather than an add-on, and recognition of frontline mediation as essential system work rather than residual support. While this study is situated in Tyrol and the Austrian healthcare system, the underlying mechanism is likely transferable to other corporatist and universal systems facing similar combinations of capacity constraints and digital transformation. What is context-specific are institutional configurations and geography; what appears generalizable is the conditional nature of digital efficiency under scarcity–that is, efficiency gains materialize only where individuals and institutions possess the resources to convert them into access (15, 84).

### 5.4 Strengths, limitations, and future research

The study’s strengths include its large qualitative sample, extended observation period, and systematic triangulation across social positions, capturing perspectives from participants across differing levels of access resources as well as institutional viewpoints. This design enabled the identification of relational and institutional mechanisms–particularly the interaction of digitalization and waiting times–that would likely remain obscured in single-group or short-term studies. The temporal span of the material further made it possible to distinguish between acute pandemic-related disruptions and the post-pandemic persistence of digitally mediated access barriers, thereby strengthening the analysis of how crisis conditions accelerated but did not fundamentally resolve existing inequalities.

Several limitations should nonetheless be acknowledged. First, recruitment partly relied on gatekeepers within social service organizations. Although this was complemented by snowball sampling and outreach in low-threshold settings, individuals who are entirely disconnected from institutional support structures may remain underrepresented. This is particularly relevant when studying digital exclusion, as those most affected may also be hardest to reach. Second, data collection spanned different stages of the COVID-19 pandemic and the subsequent years (2020–2025). While this temporal heterogeneity was analytically valuable in revealing how crisis conditions accelerated and normalized digitally mediated access arrangements, the findings still reflect evolving configurations rather than a single stable system state. The study therefore captures processes of transformation and persistence, which strengthens interpretive insight but limits claims about temporal sequencing, prevalence, or causal direction. Third, as a qualitative study, the analysis cannot quantify the magnitude of observed effects or establish causal relationships between digital exclusion, waiting times, and health outcomes. Participants’ accounts provide strong evidence of perceived mechanisms–such as delayed care, foregone services, and increased administrative burden–but these pathways cannot be directly linked to measurable health indicators within the present design.

Finally, the prominent role of intermediaries–frontline workers, family members, and informal supporters–raises questions of mediation and positionality. While such support enables access, it may also obscure the full extent of exclusion. Rather than a bias to be eliminated, this mediation is itself part of the access regime under digitalization, but it complicates attribution of barriers solely to individual capacities or system design. Future research could extend these findings through mixed-methods approaches that link qualitative analyses of navigation practices to quantitative indicators such as waiting times, service utilization, or interrupted benefit claims. Longitudinal designs, including qualitative or mixed-methods panel studies, could further illuminate trajectories of adaptation, disengagement, or cumulative disadvantage, though such designs must carefully address attrition among highly precarious populations. Before large-scale empirical extensions, systematic or scoping reviews could help consolidate evidence on digitalization as a social determinant of health, clarifying where robust quantitative findings exist and where qualitative insights remain essential. Finally, intervention-oriented research evaluating supported digital navigation, multi-channel access models, or accessibility-by-design initiatives would be valuable, particularly if such studies examine both user outcomes and organizational capacity. Comparative research across healthcare systems could further assess the transferability of the mechanisms identified beyond the Austrian context.

## 6 Conclusion

This study shows that digitalization in a near-universal system under sustained capacity constraints is not a neutral efficiency upgrade, but a key determinant of how Universal Health Coverage is realized in practice. By reshaping how access is navigated, digitalization turns digital navigation into a determinant of time-to-care and, in doing so, redefines equity in access. Across participants experiencing access vulnerability, contrast group participants with comparatively high access resources, and healthcare experts, the findings reveal a double barrier in which long waiting times increase the value of rapid information and flexible scheduling, while unequal digital prerequisites determine who can convert these opportunities into timely care. The temporal comparison further suggests that the COVID-19 pandemic did not create these dynamics anew, but exposed, accelerated, and normalized them by making digital access temporarily indispensable while leaving underlying inequalities in resources, literacy, accessibility, and institutional support largely intact. The result is stratified waiting and cumulative disadvantage within the same formal system of entitlement. Because digital barriers also shape welfare claims, education, and mental health support, digital exclusion operates as a social determinant of health through delayed care, interrupted benefits, and administrative stress. For Universal Health Coverage, the implication is immediate: coverage alone cannot secure equity when access is organized through waiting and digital self-service. Equity-oriented digital transformation therefore requires multi-channel access, supported navigation, accessibility-by-design, and institutional capacity for mediation and staff training. Without these safeguards, digitalization risks converting formal universality into stratified access by shifting the costs of scarcity management onto those least able to bear them.

## Data Availability

The datasets presented in this article are not readily available due to data confidentialty and participant anonymity. Further inquiries can be directed to the corresponding author.
